# Cooperative Evolution of China’s Excellent Innovative Research Groups from the Perspective of Innovation Ecosystem: Taking an “Environmental Biogeochemistry” Research Innovation Group as a Case Study

**DOI:** 10.3390/ijerph182312584

**Published:** 2021-11-29

**Authors:** Jie Gao, Shu Liu, Zhijian Li

**Affiliations:** 1 School of Innovation and Entrepreneurship, Shandong University, Qingdao 266237, China; littlehope@sdu.edu.cn; 2 Institute for Intelligent Society Governance, Tsinghua University, Beijing 100084, China; 3 School of Environment, Tsinghua University, Beijing 100084, China

**Keywords:** innovative research group, innovation ecosystem, cooperation network, environmental biogeochemistry, scientometrics, knowledge map, exploratory case study

## Abstract

Research, understanding, and prediction of complex systems is an important starting point for human beings to tackle major problems and emergencies such as global warming and COVID-19. Research on innovation ecosystem is an important part of research on complex systems. With the rapid development of sophisticated industries, the rise of innovative countries, and the newly developed innovation theory, innovation ecosystem has become a new explanation and new paradigm for adapting to today’s global innovation cooperation network and the scientific development of complex systems, which is also in line with China’s concept of building an innovative country and promoting comprehensive innovation and international cooperation with scientific and technological innovation as the core. The Innovative Research Group at Peking University is the most representative scientific and technological innovation team in the frontier field of basic research in China. The characteristics of its organization mechanism and dynamic evolution connotation are consistent with the characteristics and evolution of innovation ecosystem. An excellent innovative research group is regarded as a small innovation ecosystem. We selected the “Environmental Biogeochemistry” Innovation Research Group at Peking University as a typical case in order to understand and analyze the evolution of cooperation among scientific and technological innovation teams, improve the healthy development as well as internal and external governance of this special small innovation ecosystem, promote the expansion of an innovation team cooperation network and the improvement of cooperation quality, promote the linkage supports of funding and management departments, and improve their scientific and technological governance abilities. Through scientometrics, visual analysis of knowledge maps, and an exploratory case study, we study the evolution process and development law of team cooperation. It is found that the main node authors of the cooperation network maintain strong cooperation frequency and centrality, and gradually strengthen with the expansion of the cooperation network and the evolution of time. Driven by the internal cooperative governance of the team and the external governance of the funding and management departments, this group has gradually formed a healthy, orderly, open, and cooperative special innovation ecosystem, which is conducive to the stability and sustainable development of the national innovation ecosystem and the global innovation ecosystem.

## 1. Introduction

Global warming, the COVID-19 epidemic, earthquakes, ecological destruction, sudden and extreme events, and their accompanying social and economic disasters pose severe challenges to the sustainable development of mankind. However, due to the complex structure of the Earth system and the many nonlinear interactions, there are significant difficulties and obstacles for individuals aiming to understand and to predict emergencies and disasters under these complex systems; nevertheless, research on complex systems in the scientific community has not stopped [1,2,3]. The Nobel Prize in Physics, in 2021, was also awarded to research on complex systems. The study of complex systems is a comprehensive and interdisciplinary subject that includes Earth System Science (ESS) on the complex Earth system; research on climate systems, earthquake systems, environmental ecosystems, etc.; and a series of other branches of interdisciplinary scientific research, such as transplanting the concept of the environmental ecosystem into innovation research and management research, and developing the theoretical system of the innovative ecosystem [1,4,5,6,7].

With regard to the rise and development of research on innovation ecosystem, because of the rise of Silicon Valley and the success of high-tech companies such as Apple Inc., Cupertino, CA, USA, marked by two scientific and technological innovation and research reports of the President’s Committee of Advisors on Science and Technology (PCAST) in 2004, some researchers believe innovation ecosystem has been formally put forward as the core concept and normative content, and therefore have focused their attention on innovation ecosystem. It is believed that the leading position of the United States in science and technology, economy, and politics in the world mainly depends on an “innovation ecosystem” which is dynamic, evolving, and has a good top-down design [8,9,10]. Subsequently, more and more studies on the concept and theoretical practice of innovation ecosystem have appeared in the research reports and academic studies of the United States, Japan, OECD countries, and many developing countries [11,12,13,14]. Among these studies, studies on enterprise innovation ecosystem were the most common research focus in academic circles, followed by industrial and national innovation research, and of course, some studies on environment and urban planning were also involved [15,16,17]. However, research on China’s innovation system has just started, and the frequently cited and influential studies have mostly summarized the theoretical evolution and connotation characteristics of the innovation ecosystem [5,6,7,18], while other studies have mainly focused on the innovation research of enterprises and industries [16,17,19], and there are limited studies on innovation ecosystem related to scientific and technological innovation and basic research [20].

In the 21st century, China pays special attention to the construction of an innovative country. Recently, China has been focusing on the innovation-driven development strategy, accelerating the overall innovation with scientific and technological innovation as the core, and constantly advancing the modernization of the national governance system and governance capacity. Facing the changes in international trade and economic situations and the impact of the COVID-19 epidemic, China, as a big economy and complex innovation ecosystem, has achieved relatively good and stable responses and development. Some scholars believe that the development and connotation of innovation ecosystem theory are highly consistent with China’s innovation-driven development strategy and the concept of accelerating comprehensive innovation with scientific and technological innovation as the core [4,5,6,7,21,22,23].

The source and production of scientific and technological innovation are basic research. Most basic research requires promotion of the state, inclination of science and technology policies, and support of science funds. Systematic and cooperative innovation needs to be completed through a scientific and technological innovation team. From a macro perspective, the national innovation system and innovative country are large innovation ecosystems, while from a micro perspective, industries, organizations, enterprises, and innovation teams are also small- and medium-sized innovation ecosystems. Some studies on innovation ecosystem have focused on regions and regional perspectives, such as the global innovation system, national innovation system, and regional innovation system [14,15,24]. There are also studies from the perspectives of industry and business systems, and platform development [13,15,25,26,27], but there are few studies on basic research or scientific and technological innovation teams from the micro perspective and innovation ecosystem perspective. From the perspective of research methods, research on innovation ecosystem has mostly been based on single-case exploration and multi-case comparative studies [12,13,14,15,16,17]. At the same time, there have been many empirical studies based on hypothesis analysis and many studies on connotation framework [4,5,6,7,18,19,20,21,22,23,24,25,26]. Some scholars suggest using the TRIZ theory and method in the analysis of regional ecosystem theoretical model and so on [28,29,30,31,32,33,34]. Among them, the TRIZ theory and method system were summarized and proposed by Soviet scientists in their studies on patent analysis and innovation, and the subsequent development and gradual transition and enriched from the classic TRIZ to the modern TRIZ theory [28,29,30,31]. The common innovative problem-solving tools of the classic TRIZ theory include technological evolution trend, conflict analysis and invention principle, Ariz algorithm, etc., while the later developed modern TRIZ theory added benchmarking analysis, feature transfer, function-oriented search, function analysis and cutting method, root cause analysis, failure prediction analysis, and many other methods and tools [28,29,30,31,32,33,34,35,36,37]. The early classic TRIZ theory focused on how to solve invention problems, while the later developed modern TRIZ theory has been better at analyzing and discovering key problems [28,29,30,31,34,35,36,37]. Currently, research on the TRIZ theory is also combined with various new algorithms, new theories, and new methods for model design, science and technology evaluation, case reasoning, etc., and its application in the research on innovation ecosystem has also begun to appear [28,32,33,34,35,36,37]. This study mainly uses the mainstream exploratory case study method for the research on innovation ecosystem, coupled with the scientometric method and visual analysis of knowledge maps, to understand the cooperative evolution process of an innovation team, especially to show the evolution and development of an innovation cooperation network with knowledge maps. 

According to existing studies, the innovation ecosystem has time series, self-organization, and community characteristics [4,6,7,8]. From the perspective of innovation ecosystem, in this study, we select an innovative research group funded by the National Natural Science Foundation of China (NSFC), a typical representative of China’s high-level basic research science and technology innovation team, and specially selects the excellent “Environmental Biogeochemistry” Innovation Research Group at the Peking University as a typical case. The objectives and significance of this study are as follows: (1) Regarding the innovation research group as a small innovation ecosystem, we analyze the development and evolution of the cooperative innovation network and time sequence of the innovation team funded by the Department of Earth Sciences of the NSFC, and explore its internal and external interaction and cooperation laws, which can promote relevant innovation teams and small innovation ecosystems to timely understand and adjust their evolution model of cooperation and governance structure, and to contribute to the sustainable development of the team and the improvement of their internal governance ability. (2) The funding and management departments can also timely understand the development of relevant teams and innovation ecosystems through investigation and research, data analysis, and visual analysis of knowledge maps, and therefore promote linkage supports between the relevant departments and improve the ability of science and technology governance. (3) This study provides a new theoretical perspective and analysis method for the development of China’s high-level scientific and technological innovation team, contributes to the construction and development of science and technology governance system with Chinese characteristics and national innovation ecosystem, enriches innovation theory, and provides typical cases and reference practice for the development of scientific and technological innovation teams and the construction of the global innovation ecosystem.

## 2. The Cooperation Network and Time Sequence Evolution of the Innovative Research Group from the Perspective of Innovation Ecosystem

### 2.1. The Innovative Research Group as an Innovation Ecosystem

Scholars from various countries have many definitions and discussions on the connotation of “innovation ecosystem” [3,4,5,6,7,21,22,23]. Combined with the theme of a cooperation network and the time-series evolution of a scientific and technological innovation and research community, in this study, we believe that innovation ecosystem refers to a dynamic system and cooperation network that gradually integrates various resources in the time-series evolution of similar ecosystems within an interval or organization, among members of each innovation community, and between the innovation community and external environment based on a common vision and cooperation goal, and forms a symbiotic competition and cooperative evolution through the interactive transmission of information flow and energy flow.

According to the relevant regulations of the National Natural Science Foundation of China (NSFC), an “Innovative Research Group” refers to “a research group with excellent middle-aged and young scientists as academic leaders and the backbone of research, jointly carrying out innovative research around an important research direction, and cultivating and bringing up a research group that occupies a place in the forefront of international science [38]. An innovative research group is a basic research science and technology innovation team funded by the NSFC, and it is the most representative research group of scientists who advocate cooperative innovation and frontier exploration in China. This study takes it as its representative to explore the micro-level science and technology innovation ecosystem.

An innovative research group takes universities, scientific research institutes, and other institutions as supporting units, and the academic leaders, research backbone, and formal team members must belong to the supporting units, which are regional, interval, and community to a certain extent; meanwhile, the assessments of an innovative group advocate interdisciplinary research, frontier exploration, and international cooperation, and the team is also open and innovative. The members of an innovative research group have information communication and close cooperation; in addition to the formal internal cooperation network, they also cooperate with other high-level scholars or groups, which will gradually evolve into an increasingly expanding cooperation network and ecosystem during the progress of a project and subsequent development and evolution. Therefore, an innovative research group has the basic characteristics of an innovation ecosystem, which is itself an innovative cooperation network and an open and complex system that is constantly evolving in time sequence. It is of necessary theoretical and practical significance to explore the regular contents of its cooperation network and time-series evolution from the perspective of innovation ecosystem.

### 2.2. The Exploratory Case of a Cooperation Network and the Time Sequence Evolution of an Excellent Innovative Research Group

#### 2.2.1. Methodology: Exploratory Case Study and Visual Analysis of Knowledge Maps

According to many scholars’ studies and research on the development and evolution of innovation ecosystem [4,18], research on innovation ecosystem is still in the initial stage of development and construction, and the case study method is more suitable for the construction and expansion of new research fields and paradigms and is the main research method of innovation ecosystem research [25,26,27]. Earlier, Eisenhardt (1989) proposed that case studies should construct and develop theories and explore new fields and paradigms [19]. Subsequently, Eisenhardt and his collaborators also paid more attention to the theory and practice of micro-foundations, i.e., small organization systems, and team development in dynamic environments [18]. Some scholars have also conducted benchmarking analysis, feature transmission, and case reasoning research on regional innovation ecosystem or specific innovation problems based on TRIZ-related theories and methods [31,32,33,34]. However, this study mainly analyzes the governance evolution mechanism and the development of innovation cooperation networks of an innovative research group. We conducted research and interviews with the National Center for Science and Technology Evaluation (NCSTE) and the National Natural Science Foundation of China (NSFC) on relevant research objectives and research contents. Therefore, this study mainly adopts an exploratory case study.

As the case study and evolution analysis involve the period presentation and analysis of innovation cooperation network, as well as the cooperation frequency and network centrality of the main node authors, these all need to be analyzed with the methods of scientometrics and visual analysis of knowledge maps. Scientometrics (Scientific Metrology) has a wide connotation and scope, including citation analysis, bibliometrics, etc. that were widely used in the early stage, and visual analysis of knowledge maps, knowledgometrics, altmetrics, etc. that were developed in the later stage [39,40]. A knowledge map is an image that shows the relationship between the development process and structure of scientific knowledge with the knowledge domain as the object. CiteSpace visualization software is a representative tool of scientometrics and knowledge map visualization, which can draw the knowledge map of scientific cooperation networks and calculate the key information of main node authors or clusters of each network, such as network density, cooperation frequency, and network centrality [39,40,41,42,43,44,45]. Recently, based on scientometrics and knowledge map visual analysis, research on innovation teams has gradually increased [40,46], but most studies have focused on the macroscopic discussion of knowledge base and discipline frontier or the analysis of the relationship between authors’ citations and co-citations, and the results of direct research on cooperation among authors and other analysis indicators of scientometrics (such as network density and centrality) are few. Therefore, in this study, it is included in the case study and evolution analysis of a typical excellent innovation team. Using the methods of scientometrics, visual analysis of knowledge maps, and a case study, combined with survey materials and paper data downloaded from the database Web of Science Core Collection, we used software to draw the knowledge map of an innovation cooperation network in each stage of evolution, we calculated the cooperation frequency and network centrality of the main node authors, and we assisted the case study to better present the cooperative evolution of the innovative research group. The figures of this study were drawn and calculated by the authors using software.

In order to deeply analyze the cooperation mechanism and time sequence evolution of an innovation group cooperation network from the perspective of innovation ecosystem, we selected the excellent Environmental Biogeochemistry” Innovation Research Group of the Earth Sciences Department of the NSFC for the exploratory case analysis, supplemented by scientometrics and visual analysis of knowledge maps, and therefore clearly show the process and connotation of the cooperation network and time sequence evolution.

According to our previous research on innovative research groups [40,41,47,48], combined with the materials provided by the National Center for Science and Technology Evaluation (NCSTE) and the National Natural Science Foundation of China (NSFC), as well as our later supplementary research and interviews, we can understand that the project integrates environmental science, bioscience, material chemistry, toxicology, and other disciplines. Its research direction is environmental biogeochemistry, focusing on the circulation, behavior, fate and effects of carbon, nitrogen, persistent toxic pollutants, and endocrine disruptors in the environment. It has the cutting-edge characteristics of basic research; in addition, it also meets the interdisciplinary requirements advocated by the innovative research group project, and has great potential for discipline construction and innovation breakthrough, with outstanding representativeness. Meanwhile, this group is an innovative group led by S. Tao, a famous environmental geographer in China and academician of the Chinese Academy of Sciences. It has been supported for 6 years before and after, and the project performance evaluation is “excellent”. The research results and personnel training effects are remarkable, and in addition to being published in academic journals such as Nature and Science, they have also won various scientific and technological awards many times; the academic leader was elected as an academician of the Chinese Academy of Sciences shortly after the end of the project, and another academic backbone became an academician of the Chinese Academy of Sciences during the implementation of the project. At the same time, many other academic backbones and member groups were supported by the “National Outstanding Youth Fund” or selected into the “New Century Excellent Talents Plan” and other honors. In 2011, the project also participated in the international evaluation of the 25th anniversary of NSFC as a typical case. Therefore, combined with the materials of this team and the materials of our supplementary investigation and interview, through the knowledge map visual analysis, scientometrics, and exploratory case analysis, we explore the development, evolution, and connotation of its cooperation network from the perspective of innovation ecosystem.

During the research period of the project, from 2001 to 2006, 183 SCI documents were retrieved from the academic achievements of the main backbone members. Three years after the completion of the project, from 2007 to 2009, 116 documents were retrieved. Through scientometrics and knowledge map visual analysis, using CiteSpace software, we describe the time sequence evolution of cooperation, distribution, and clustering of main node authors [39,40,41,42,43,44,45], and conduct an exploratory case analysis of the “Environmental Biogeochemistry” Innovative Research Group, with S. Tao as the project leader from multiple dimensions.

#### 2.2.2. Research Period of the Project Implementation (2001–2006)

With the support of the NSFC, the innovative reasearch group invested eight researchers during the project implementation period from 2001 to 2006, with S. Tao as the academic leader and J.-H. Fang, J.-Y. Hu, and X.-J. Wang as the research backbones. The eight members of the team included two academicians of the Chinese Academy of Sciences (one member was selected during the project implementation and one member was selected after the project conclusion) and five members were winners of the National Science Fund for Distinguished Young Scholars (three members were funded before the project was launched and two members were funded after the project was completed), two members were selected into the New Century Excellent Talents Plan of the Ministry of Education of China (one member during the project implementation and one member after the project was completed). It can be seen that this group, as an innovation ecosystem, has achieved good research results and cultivated and expanded the team of research talent in the expansion and time evolution of the cooperation network.

According to the interview, report materials, and previous studies [40,41,47,48], the research direction of this group is environmental biogeochemistry, focusing on the circulation, behavior, fate, and effects of carbon, nitrogen, persistent toxic pollutants, and endocrine disruptors in the environment. The purpose was to put forward the continuous biomass conversion factor method through interdisciplinary research, to estimate the carbon storage and its temporal and spatial variation law of terrestrial ecosystem in China, and to re-analyze the carbon storage of forest vegetation in the northern hemisphere. The quantitative method was followed for studying the fate and effects of regional persistent organic pollutants from emission estimation, multi-media source analysis, fate simulation, exposure simulation to ecosystem, and human health risk analysis. In addition, the environmental behaviors and impacts of polycyclic aromatic hydrocarbons and organochlorine pesticides were deeply studied; the behavior, fate, and ecological effects of endocrine disruptors were systematically studied in typical polluted areas in China.

During the implementation of the project, this group made a series of breakthrough achievements in the related attributes of the terrestrial ecosystem carbon cycle and its response to climate change, the regional environmental process of persistent organic pollutants, and the environmental fate and ecological effects of endocrine disruptors, etc. For example, the research group perfected the application of the “Continuous Biomass Conversion Factor Method” proposed earlier in a regional scale carbon assessment and studied the carbon budget of Japanese vegetation by using this method, which solved the dynamic change problem of the Japanese carbon budget that Japanese scholars had not solved for many years. The ecological risk assessment method with the expected loss of population sustainability as the evaluation endpoint was established, and the ecological risk of the Taihu Lake Night Heron population caused by DDE exposure was evaluated. Many original achievements have been published in the world’s top journals such as *Science*, *Nature*, and *Environmental Science & Technology*, which has made the group an internationally renowned academic group engaged in environmental biogeochemistry research [41].

**Figure 1:**
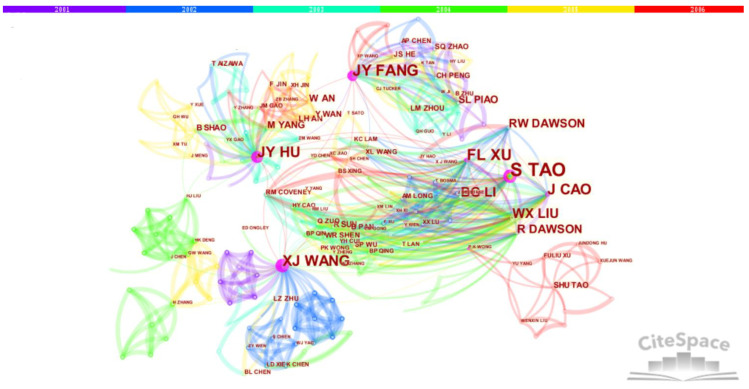
The knowledge map of the SCI paper cooperation network during the research period of the “Environmental Biogeochemistry” Innovative Research Group’s projects (2001–2006) (node type).

**Figure 2:**
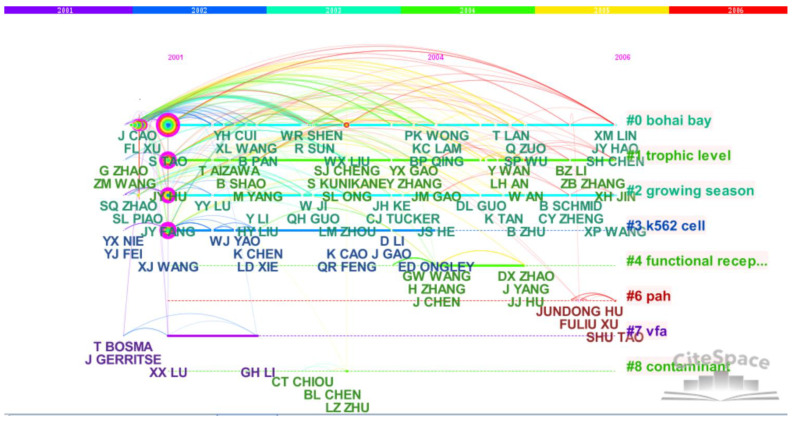
The knowledge map of the SCI paper cooperation network during the research period of the “Environmental Biogeochemistry” Innovative Research Group’s projects (2001–2006) (timeline type).

We drew the paper cooperation network knowledge maps (node map and timeline map) of the main group members during the project implementation period, as shown in Figure 1 and Figure 2, respectively. According to scientometrics and CiteSpace software, we obtained the relevant information of the main node authors of the cooperation network, as shown in Table 1.

It can be seen from Figure 1 and Figure 2 that S. Tao, the academic leader, has the strongest cooperation with J.-Y. Fang, F.-L. Xu, X.-J. Wang, J.-Y. Hu and others, which runs through the whole project. Among overseas scholars, he cooperates most with R. W. Dawson. It can be seen from the timeline knowledge map (Figure 2) that paper cooperation with S.-L. Piao and W.-X. Liu began to appear from 2003 to 2004, followed by the addition and growth of many new team members or academic rookies such as Y. Yang and Z.-Y. Tang, and the cooperation network continues to expand and evolve. It can be seen from the maps (Figure 1 and Figure 2) and Table 1 that S. Tao, the academic leader, has the strongest network centrality (the thickest purple circle and the highest centrality value of 0.97) and the highest cooperation frequency as compared with others. The second place in cooperation frequency and network centrality is J.-Y. Fang, an academician of the Chinese Academy of Sciences during the project implementation. Many important achievements, such as articles published in *Nature* and *Science* journals, were completed by cooperation with team members and foreign scholars during the project research. Other backbone members, such as F.-L. Xu and J.-Y. Hu, also have high cooperation frequency and network centrality, and each member has their own subgroup and network cluster.

During the research period of the project, the five representative papers of this group are shown in Table 2.

#### 2.2.3. Three-Year Development Period after the Completion of the Project (2007–2009)

On the basis of the results achieved during the implementation of the project, the group members carried out further research after the conclusion of the project and achieved remarkable results [48]. For example, the distribution and possible causes of carbon sources and sinks in China’s terrestrial ecosystem were systematically analyzed, and the temporal and spatial pattern and mechanism of China’s terrestrial carbon sinks and carbon sources were comprehensively studied. It was preliminarily estimated that the CO_2_ absorbed by China’s land could offset the share of CO_2_ emissions from industrial sources, providing a relevant reference and a basis for China’s relevant departments to formulate energy conservation and emission reduction policies and for China’s international negotiations on climate change. By cooperating with botanists from all over the world, the team members have established a distribution database of Chinese woody plants containing more than 11,000 species. It was found that the number of species in China and North America was indeed limited by energy, as predicted by the “metabolic theory”, but the size of spatial scale greatly affected the relationship between energy and diversity. The first global inventory of PAHs emissions was established and published. The concept of gene susceptibility was quantitatively introduced into exposure and risk simulation, which quantitatively confirmed that polycyclic aromatic hydrocarbons were an important pollutant inducing lung cancer in Chinese residents. A multicomponent method for the determination of trace estrogen, androgen, progesterone, glucocorticoid, and mineralocorticoid in the environment was established. It was found that there were serious deformities in the juvenile of wild Chinese sturgeon in the Yangtze River, and it was proven that triphenyltin was the main substance causing the distortion of wild Chinese sturgeon.

The five representative papers in the three years from 2007 to 2009 after the project completion are shown in Table 3.

**Figure 3:**
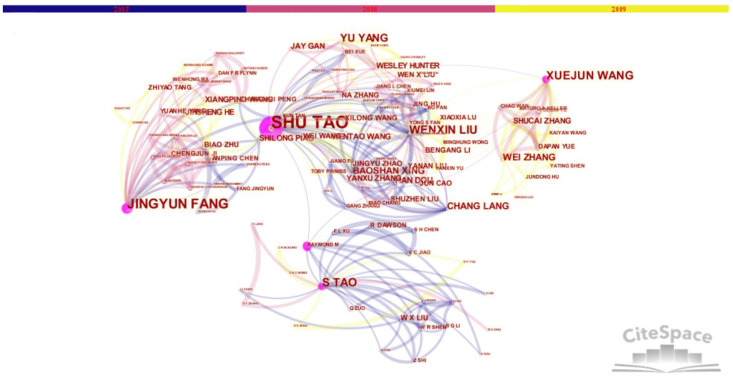
The knowledge map of the SCI paper cooperation network after the completion of the “Environmental Biogeochemistry” Innovative Research Group’s projects (2007–2009) (node type).

**Figure 4:**
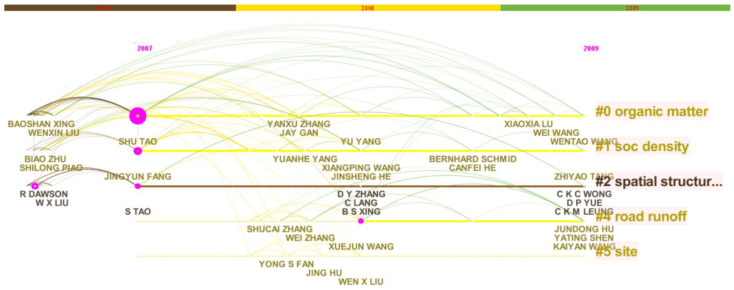
The knowledge map of the SCI paper cooperation network after the completion of the “Environmental Biogeochemistry” Innovative Research Group’s projects (2007–2009) (timeline type).

We drew the knowledge maps of the SCI papers cooperation network of major members of the “Environmental Biogeochemistry” Innovation Research Group from 2007 to 2009, as shown in Figure 3 and Figure 4. According to scientometrics and CiteSpace software, we obtained the relevant information of the main node authors of the cooperation network, as shown in Table 4.

It can be seen from Figure 3 and Figure 4 that the cooperation and distribution of the node authors are still dominated by the academic leader S. Tao and scientific research backbones J.-Y. Fang, X.-J. Wang, etc. At this time, two members have become academicians of the Chinese Academy of Sciences, and many members have won the National Outstanding Youth Fund and other projects or honors. According to the maps and Table 4, there are some relatively new node authors with more cooperation frequency or certain centrality, such as Y. Yang and others in China, and R. Dawson and others in foreign countries. In the three years after the completion of the project, the team’s annual cooperative publication volume has further increased rapidly, and the innovation cooperation network has further expanded.

According to the interview, report materials and previous studies [40,41,47,48], the papers published on PNAS in cooperation with European and American scientists have attracted wide attention, and more than 300 media outlets around the world, including *Science*, reported it. The “Continuous Biomass Conversion Factor Method”, which was proposed to calculate regional forest biomass, has been used as a general method and is widely used. The multi-media source analysis method established by the research group has been praised by peers as “an important contribution to our understanding of pollutant sources and environmental behaviors, and represents “culmination” in the aspects of source characterization of polycyclic aromatic hydrocarbons, analysis of a large number of samples, application of fugacity model, statistical analysis and sensitivity, and uncertainty analysis. Papers on the county-level resolution emission list of polycyclic aromatic hydrocarbons in China were listed as the highest cited papers of the year after *Environmental Science & Technology* was published. The quantitative research results of lung cancer risk caused by respiratory exposure to polycyclic aromatic hydrocarbons in China are listed as important articles in the current period by the PNAS. The direct evidence of fish distortion caused by environmental pollution has become an important reference for explaining fish distortion in wild ecosystems. The research results on steroid pollution in municipal wastewater have become an important basis for peers to carry out research on new persistent pollutants.

The research group has a certain international influence in relevant research directions. At present, many people have served in international academic organizations (members of the global terrestrial carbon observation (TCO) working group, “SETAC Asia Pacific chairman, etc.) and served as the editorial board of foreign academic journals.

### 2.3. The Network Evolution and Internal and External Governance of Innovative Research Group as Innovation Ecosystem

Through the exploratory case analysis of the cooperation network and time sequence evolution process of innovation groups, it can be found that as a small innovation ecosystem, excellent innovation groups have formed a good cooperation structure and the innovation network for their scientific research teams and research communities, academic leaders as “head geese”, and other research backbone structures is reasonable. On the basis of young scientists, combined with other researchers and postgraduates, an innovative ecosystem community was formed; at the same time, the community exchanged and cooperated with other community members individually or as a whole, and gradually expanded into a larger cooperative network, forming an open, complex, and dynamic ecosystem in the time sequence evolution.

As an innovation ecosystem, the innovative research group is also supported and funded by the National Natural Science Foundation of China (NSFC), managed and reviewed by the foundation committee and the expert review committee, and supervised and managed by the supporting units, and therefore organically combines external governance with internal cooperation of the team [48]. At this level, it also forms a systematic mechanism of collaborative integration and symbiotic evolution.

In the typical case report materials submitted to the National Natural Science Foundation of China (NSFC) and National Center for Science & Technology Evaluation (NCSTE), the group thinks that: (1) For the members of the group, the project provides relatively sufficient research funds and condensed the research direction of the team. The implementation of the talent funds has high flexibility, is not strictly limited by specific research programs, and is easy to exert initiative and creativity, which provides important conditions for young scientists to become talents. (2) For the group, the implementation of the project provides better conditions for cooperation among members, enabling them to freely cooperate, innovate, grow, and evolve [48]. Therefore, as a special and micro innovation ecosystem, an innovative research group not only needs a good free environment and platform for internal governance and cooperative evolution, but also needs the support and governance of external relevant departments and policies to form a multi-dimensional and multi-level science and technology governance system and innovation ecosystem.

## 3. Conclusions

This study is based on the perspective of innovation ecosystem and uses a case study, as the mainstream method of related research, to explore the process and connotation of the evolution of a cooperation network and time sequence of an outstanding innovation group as a special type of innovation ecosystem. It is considered that the innovative research group conforms to the connotation and characteristics of the innovation ecosystem, and has the characteristics of interval, openness, innovation, dynamics, and complexity. In the evolution and development of the cooperation network, through the guidance and drive of academic leaders and research backbones, it continues to coordinate integration and cooperative innovation, and therefore achieves a series of important research progress and talent training objectives. It has also formed an innovation ecosystem of symbiotic evolution.

Specifically, at the level of practice and case study, we select the “Environmental Biogeochemistry” Innovative Research Group, organize its development and governance context according to relevant materials and timeline, draw the knowledge maps of the scientific research cooperation network in the periods of the project implementation and completion, and analyze the characteristics of cooperation among the main node authors of the network (cooperation frequency and network centrality). According to the materials, we summarize the external management of the Science Fund for Innovative Research Group, the National Natural Science Foundation of China, team cooperation, and internal governance of innovative research groups, which can promote the expansion of the innovative research group cooperation network and time sequence evolution, and promote it to form an open and complex innovation ecosystem.

## 4. Discussion

From the Earth and the country, down to organizations, teams, and individuals, in the face of routine changes and emergencies, the concepts and methods of complex network systems help to achieve stability and sustainable development. From a macro perspective, in the period of COVID-19, China’s economic development, technological innovation, international cooperation, health of people, and stability of working life all benefited from the construction of a good innovation ecosystem. At the same time, China has actively strengthened international cooperation in scientific and technological innovation and public health, actively integrated into the world innovation cooperation network, nested and integrated the national innovation ecosystem with the global innovation ecosystem, and symbiotically evolved.

Meanwhile, at the micro level, as a special small innovation ecosystem, science and innovation teams such as innovative research groups also need to form an open and expanded innovation cooperation network and ecosystem that is nested, linked, and integrated into larger science and technology organizations and innovation ecosystems of industry, country, and the world under the collaborative promotion of internal cooperation governance of the team and external governance of relevant departments. As a result, from the micro to macro level and from the inside to outside multi-layer linkage, science and innovation teams could truly achieve comprehensive innovation, and promote the development of global innovation ecosystem, to effectively respond to global warming, economic crises, COVID-19, and other major challenges and unexpected events.

## Figures and Tables

**Table 1:** Relevant information of the main node authors of the SCI paper cooperation network during the research period of the “Environmental Biogeochemistry” Innovative Research Group’s projects with significant cooperation frequency and centrality.

Frequency	Centrality	Main Node Author
93	0.97	Tao, S.
57	0.63	Fang, J.-Y.
42	0.19	Wang, X.-J.
39	0.33	Hu, J.-Y.
36	0.28	Xu, F.-L.
31	0.14	Cao, J.
29	0.11	Liu, W.-X.

**Table 2:** The information of representative papers during the research period of the project.

	Thesis Title	Authors	Journal, Time, and Volume
1	Changes in forest biomass carbon storage in China between 1949 and 1998	Fang, J.-Y.; Chen, A.-P.; Peng, C.-H.; Zhao, S.-Q.; and Ci, L.	*Science*, 22 June 2001, Vol. 292, Issue 5525, pp. 2320–2322 [49]
2	Interannual variability in net primary production and precipitation	Fang, J.-Y.; Piao, S.-L.; Tang, Z.-Y.; Peng, C.-H.; and Ji, W.	*Science*, 7 September 2001, Vol. 293, Issue 5536, p. 1723 [50]
3	Organochlorine pesticides in agricultural soil and vegetables from Tianjin, China	Tao, S.; Xu, F.-L.; Wang, X.-J.; Liu, W.-X.; Gong, Z.-M.; Fang, J.-Y.; Zhu, L.-Z.; and Luo, Y.-M.	*Environmental Science & Technology*, 2005, Vol. 39(8): 2494–2499 [51]
4	A chemical extraction method for mimicking bioavailability of polycyclic aromatic hydrocarbons to wheat grown in soils containing various amounts of organic matter	Tao, S.; Xu, F.-L.; Liu, W.-X.; Cui, Y.-H.; and Coveney, R.-M.	*Environmental Science & Technology*, 2006, Vol. 40(7): 2219–2224 [52]
5	Dispersion modeling of polycyclic aromatic hydrocarbons from combustion of biomass and fossil fuels and production of coke in Tianjin, China	Tao, S.; Li, X.-R.; Yang, Y., Coveney, R.M.; Liu, X.-X.; Chen, H.-T.; Shen, W.-R.	*Environmental Science & Technology*, 2006, Vol. 40(15): 4586–4591 [53]

**Table 3:** The information of representative papers in the period after the project completion.

	Thesis Title	Authors	Journal, Time and Volume
1	Net carbon dioxide losses of northern ecosystems in response to autumn warming	Piao, S.-L.; Ciais, P.; Friedlingstein, P.; Peylin, P.; Reichstein, M.; Luyssaert, S.; Margolis, H.; Fang, J.-Y.; Barr, A.; and Chen, A.-P.	*Nature*, 3 January 2008, Vol. 451, Issue 7174, p. 49–52. [54]
2	The carbon balance of terrestrial ecosystems in China	Piao, S.-L.; Fang, J.-Y.; Ciais, P.; Peylin, P.; Huang, Y.; Sitch, S.; and Wang, T.	*Nature*, 23 April 2009, Vol. 458, Issue 7241, p. 1009–1013. [55]
3	Temperature dependence, spatial scale, and tree species diversity in eastern Asia and North America	Wang, Z.-H.; Brown, J.H.; Tang, Z.-Y.; and Fang, J.-Y.	Proceedings of the National Academy of Sciences of the United States of America, 2009, 106(32): 13388–13392. [56]
4	Inhalation exposure to ambient polycyclic aromatic hydrocarbons and lung cancer risk of Chinese population	Zhang, Y.-X.; Tao, S.; Shen, H.-Z.; and Ma, J.-M.	Proceedings of the National Academy of Sciences of the United States of America, 2009, 106(50): 21063–21067. [57]
5	Sorption and Competition of Aromatic Compounds and Humic Acid on Multiwalled Carbon Nanotubes	Wang, X.-L.; Tao, S.; and Xing, B.-S.	*Environmental Science & Technology*, 2009, Vol. 43(16): 6214–6219. [58]

**Table 4:** Relevant information of the main node authors of the SCI paper cooperation network after the completion of the “Environmental Biogeochemistry” Innovative Research Group’s projects with significant cooperation frequency and centrality.

Frequency	Centrality	Main Node Author
54	0.98	Tao, S.
37	0.78	Wang, X.-J.
32	0.65	Fang, J.-Y.
30	0.62	Yang, Y.
11	0.32	Dawson, R.
